# Skype Supervised, Individualized, Home-Based Rehabilitation Programs for Individuals With Rett Syndrome and Their Families – Parental Satisfaction and Point of View

**DOI:** 10.3389/fpsyg.2021.720927

**Published:** 2021-09-16

**Authors:** Meir Lotan, Elena Ippolito, Martina Favetta, Alberto Romano

**Affiliations:** ^1^Department of Physical Therapy, School of Health Sciences, Ariel University, Ariel, Israel; ^2^Israeli Rett Syndrome National Evaluation Team, Sheba Hospital, Ramat-Gan, Israel; ^3^SMART Learning Center, Milan, Italy; ^4^Motion Analysis and Robotics Laboratory, Unit of Neurorehabilitation, Department of Neuroscience, Bambino Gesù Children’s Hospital, Rome, Italy; ^5^Centro AIRETT Ricerca e Innovazione (CARI), Research and Innovation Airett Center, Verona, Italy

**Keywords:** Rett syndrome, telerehabilitation, personal satisfaction, exercise therapy, parents, home exercise program

## Abstract

Individuals with Rett syndrome (RTT) experience impaired gross motor skills limiting their capacity. Therefore, they need support to participate in physical activities, and it is crucial to work with primary caregivers when developing appropriate strategies, thereby leading to an active lifestyle. There is limited evidence supporting the effectiveness of remotely supported physical activity interventions. This project aimed to evaluate the effects of a skype-based, telehealth-delivered physical activity program carried out by participants’ parents at home. This article will focus on parental points of view. A mixed-methods design evaluating parental satisfaction was conducted. Forty participants with a confirmed genetic diagnosis of RTT and their families were recruited. The intervention included a 12-week individualized daily physical activity program carried out by participants’ parents and bi-weekly supervised by expert therapists. Parents’ impressions and feelings related to the program implementation were collected throughout semi-structured interviews, and an *ad hoc* developed questionnaire and discussed. The current project results suggest that a remote physical rehabilitation program, supported fortnightly by video calls, represents an effective way of conducting a remote physical therapy intervention for this population and that it can be easily carried out at home by primary caregivers, promoting positive functional changes, without bringing feelings of frustration due to the required workload. The strategies that families have learned during the program to support the motor activities of their daughters represent an easily performed set of tools that they can maintain and use in everyday life even after the cessation of the program.

## Introduction

Individuals with disabilities present complex diagnostic and therapeutic challenges. These challenges require intense intervention programs (Cooley and McAllister, 2004). One such syndrome presenting complex clinical characteristics is Rett syndrome (RTT). RTT is a severe neurodevelopmental disorder characterized by losses in intellectual functioning, fine and gross motor skills, and communicative ability. Other features include deceleration of head growth and the development of stereotypic hand movements, occurring after a period of apparently normal development. Moreover, individuals with RTT often develop seizures, disturbed breathing patterns, characterized by hyperventilation and periodic apnea, scoliosis, growth retardation, apraxia, gait disturbances, and other abnormalities (Epstein, 1995). Diagnosis of RTT results from an X-linked dominant mutation in the MECP2 gene (Amir et al., 1999, 2000). To date, mutations in this gene can be found in about 90% of females presenting with the classical phenotype (Neul and Zoghbi, 2004). The estimated incidence of RTT is 10–15 per 100,000 females, although a higher incidence rate has been reported by some researchers (Pini et al., 1996; Skjeldal et al., 1997; Fombonne et al., 2003). It is considered to be the second most common cause of multiple disabilities in females resulting from a genetic disorder after Down’s syndrome (Christodoulou, 2001). Due to the rarity of RTT, those affected are scattered geographically, and local therapists typically see small numbers of cases which limits their capacity to develop expertise to provide the very best care. Families may be uncertain about strategies that could be of assistance, and therefore, guidance would be highly valued, assisting individuals with RTT to achieve maximal functional achievements. The family, as the primary caregiver, plays a vital role in ensuring the health and wellbeing of their child. Bly (when referring to small children) suggests that the more involved the family becomes, the more consistent therapeutic management becomes (Bly, 1999). Therefore, the healthcare professional must involve family members in all areas of planning, delivery, and evaluation of health and developmental services (Ramey and Ramey, 1996). Moreover, home-based intervention programs have shown positive effects on function and reduced mortality for persons with chronic diseases, and decreased stress for their caregivers (Gitlin et al., 2003). Moreover, a Cochrane review found that home-based programs appear to be superior to center-based programs in terms of adherence to exercise, especially in the long term (Ashworth et al., 2005). Results from a randomized, controlled trial of a home-based intervention program for children with developmental disability showed changes in cognitive development and behavior over time which favored the children who received the extra intervention at home. These results suggest the need for daily reinforcement of skills learned at the center-based program and the importance of involving families in the intervention programs of their children with disabilities (Rickards et al., 2009). Although not specific to a certain disability, these findings might also be meaningful for those with RTT. There is existing and accumulating evidence suggesting the effectiveness of individually tailored therapy programs on patient outcome in physical therapy (Jansson and Söderlund, 2004; Åsenlöf et al., 2005; Roche et al., 2007) as well as in other therapeutic disciplines, such as cognitive behavioral therapy (Broomfield et al., 2011; Carlbring et al., 2011; Hoogsteder et al., 2015), oral hygiene (Jönsson et al., 2009, 2010), nursing (Mertz et al., 2017), and others. This treatment philosophy is based on the concept that each client (even those with a similar diagnosis) is different from the others. Therefore, each client should be treated through an individually tailored intervention program. Such a program should be constructed based on scientific evidence, therapist experience, and client-specific needs (Makam and Nguyen, 2017). Previous findings describing the implementation of such a program with five individuals with RTT (Lotan et al., 2021) suggest that a six-month intervention program carried out by participants’ parents at home and monthly remotely supervised resulted in significant functional improvements for this group of clients with a high level of parents satisfaction. This positive atmosphere suggests the possibility of implementing a similar project with a larger sample to strengthen the evidence in this field. However, since such a program falls on the parents’ shoulders, it is essential to inspect parental satisfaction from program implementation as a key element in its success or failure. Within the last years, a growing body of literature explored parental satisfaction levels with telerehabilitation services for children with disabilities. Assenza et al. (2020) analyzed the answer of 144 caregivers of children with and different disabilities finding a medium-high range of satisfaction with the intervention. Their results suggested that young children’s parents (age 0–3years) felt overwhelmed with remote care and referred a low perception of the capability of telerehabilitation in enhancing their children’s goals. On the other hand, they reported a high perception of feeling helped in organizing daily activity. In the same study, parents of children over 6years of age referred a low perception of telerehabilitation make them feel in line with the in-person therapy plan. Moreover, caregivers under 40 showed a high probability of perceiving telerehabilitation as supportive. These findings suggest that both parents’ and children’s age could affect the satisfaction with telerehabilitation services. Furthermore, these authors (Assenza et al., 2020), as well as others (Utidjian and Abramson, 2016; Brophy, 2017; Tanner et al., 2020), reported that the level of experience in technology use could be a significant barrier to telerehabilitation, affecting the client’s satisfaction. Due to the scarcity of existing knowledge on variables affecting parental satisfaction with telerehabilitation services for children with disabilities, several articles reported a high level of parents’ satisfaction whose children received remote therapy intervention (Hinton et al., 2017; Assenza et al., 2020; Camden et al., 2020; Sanders et al., 2020; Tanner et al., 2020; Caprì et al., 2021). Although the literature in this field is growing, parental satisfaction with physical telerehabilitation services with children with severe developmental disabilities was only rarely explored and, to the authors’ knowledge, only one article explored this variable in parents of children with RTT with an exclusive focus on the quantitative point of view (Romano et al., 2021).

The current paper aims to describe parental satisfaction emerging from implementing an individually tailored, telehealth-delivered, and physical activity program for a cohort of people with RTT, carried out by participants’ parents at home from both quantitative and qualitative points of view.

## Materials and Methods

### Ethical Approval

The present research was conducted following the Declaration of Helsinki principles.

Ethical approval was achieved through the Ariel University IRB (AU-HEA-ML-20190326-1), and all parents signed an informed consent form after understanding and agreeing with the specifics of the research program.

### Participants

Power calculation analysis for sample size based on previous research assessing the response of individuals with RTT to physical activity program was conducted, suggesting a sample size of 40 participants to perform solid statistical analysis and reject the null hypothesis. As indicated by power calculation, 42 girls and women with genetically confirmed classic RTT living at home and their parents were involved in this project. Participants were recruited from the Italian Rett Association database.

### Study Design

A mixed-method design was applied, evaluating parental satisfaction from a qualitative as well as a quantitative point of view. The researchers used the Participatory Action Research (PAR) method. PAR involves clients (in this case, families and support staff of the person with RTT) throughout the research process, working as a team with the researcher to identify problems and disentangle them. The steps representing the progression of a PAR process involve numerous cycles of:

- assessment (of each participant’s therapeutic needs in this case);
- mutual goal attainment (individual therapeutic goals were constructed for each participant, together with her family, in relation to their expectation, needs, and availability within the family’s framework);
- action (the program implementation by the families);
- reflection (each program was discussed and revised bi-weekly through Skype meetings between a trained supervisor and parents); and
- evaluation (the program is modified if necessary and re-implemented).

Action research assumes that the participant’s involvement empowers them, resulting in greater welfare for the person with RTT and her family. Action researchers seek change and develop solutions in collaboration with the participant while maintaining sensitivity to the family’s needs and desires (Ozanne and Saatcioglu, 2008). PAR design has a weaker level of evidence than a randomized controlled trial. However, it is ideally suited in an evolving program within a highly personal collaborative framework and seems suitable for the current study. Moreover, the researchers would like the parents to be involved in the whole process, thereby understanding the process and eventually carry it further for the wellbeing and functional status of the participants with RTT after research termination.

### Procedure

Before the start of the intervention, informed consent was collected for all participants. Then, participants were randomly divided into two groups (Group 1 and Group 2) who followed the same procedure, starting it 6months apart from each other. All participants underwent four assessment sessions at three-month intervals (±1month, T1-T4). During each meeting, passive joint mobility and gross motor skills were assessed for each participant. The participants’ evaluations were conducted by two authors experienced in the rehabilitation of people with RTT (AR and ML). AR is an Italian developmental therapist and researcher who evaluated more than 250 girls and women with RTT around Italy and ML is a world-known researcher, consultant, and physical therapist who have seen more than 600 individuals with RTT across the globe and is working as a clinician with 12 girls with RTT on a weekly basis.

In the first meeting (T1), relevant information was collected on participants’ clinical conditions (such as epilepsy, osteoporosis, sleep disturbances, and other conditions typically associated with RTT) and on ongoing therapeutic interventions (such as physiotherapy, hippotherapy, hydrotherapy, and others), and the expectations and wishes of the parents regarding the improvement of motor function for their daughter were discussed together with researcher and referred professionals. During this meeting, a draft of the individualized rehabilitation goals for the intervention phase was discussed with participants’ parents and rehabilitation professionals. Rehabilitation goals were selected within four areas: (a) passive limb joints range of motion improvement; (b) functional motor abilities; (c) hand functioning; and (d) general physical health (weight, bone density, and heart BPM during physical effort).

No changes were made to participants’ daily activities between the first and second evaluation meetings (baseline phase).

In the second assessment/baseline session (T2), the rehabilitation goals were again discussed with each family and referred rehabilitation professionals and corrected if necessary.

Between the second (T2) and third (T3) evaluation meetings (intervention phase), for each participant, an individualized program of simple therapeutic activities was drawn up by two authors (AR and ML) and shared with the family. Such programs aimed to persecute the identified therapeutics objectives through easy physical activities to be carried out in the girl’s daily life for a total time of about one non-continuous hour a day for 5days a week. Each family was able to organize the activities during the week according to their routines and habits. The rehabilitation activities foreseen in the programs included but were not limited to: (a) maintaining passive postures to prevent the onset or worsening of musculoskeletal problems secondary to RTT; (b) maintaining active symmetrical and asymmetrical postures to rebalance the trunk muscles and improve balance; (c) exercise of residual functional skills (e.g., sitting position, standing position, walking, postural passages, and climbing/descending stairs); and (d) functional use of hands. The goals and activities identified were markedly different among participants in content, difficulty, and intensity to best suit each individual. After 2weeks from the delivery of the program, necessary for familiarization with the activities and the finding of any therapeutic material needed (e.g., rehabilitation pillows, treadmills, and others), a cycle of supervisions was started and carried out remotely every 2weeks for 1hour each through a videoconference platform (Skype) until the end of the intervention. The skype meetings were conducted by one author (AR) together with the participants and their parents and therapists (when available). The first supervision meeting was mainly used to clarify doubts relating to the practical implementation of the activities proposed in the program. The subsequent supervision meetings were aimed at supporting the execution of the programs by answering the parents’ questions, adapting the program to emerging needs, solving problems, rearranging the timetable, adapting the proposed exercises, evaluating, and sharing the achievement of objectives and, if necessary, setting new goals, following the PAR model.

At the end of the intervention phase, during the third evaluation meeting (T3), the level of achievement of rehabilitation goals was assessed, and the parents’ point of view relating to the intervention phase was collected. Within this meeting, parents’ remarks regarding the intervention difficulty were collected by two authors (EI and MF) through the use of a semi-structured interview. The above-mentioned researchers are Italian developmental therapists and researchers with prior knowledge of RTT, which introduced themselves to the parents as external professionals having no previous connection with the therapists who carried out the evaluations and the interventions. Those conversations with parents lasted for 1hour each, as time was devoted to allowing parents to acclimate and create an environment conducive to sharing the positive and negative aspects of implementing the program.

Between the third (T3) and fourth (T4) evaluation sessions (wash-out phase), the telehealth supervision meetings were suspended, and the families were informed that they could, at their discretion, continue or interrupt the program of activities.

During the fourth evaluation meeting (T4), the parents’ points of view were collected again by EI and MF using the same modalities used at T3. Moreover, each family was given a satisfaction questionnaire concerning the carried-out project to be returned *via* e-mail to EI within 1week from the end of the meeting.

### Outcome Measures

#### RTT Severity Level

The severity of clinical manifestation of RTT was assessed at T1 with the Rett Assessment Rating Scale (RARS; Fabio et al., 2005). Each item concerns a specific phenotypic characteristic and describes four increasing levels of its severity. The total score allows the evaluator to identify the level of severity of RTT, conceptualized as a continuum ranging from mild symptoms (lower score) to heavy deficits (higher score; Vignoli et al., 2010). The RARS was established by a standardization procedure involving a sample of 220 Italian patients with RTT. Internal consistency was found at 0.912, and the internal consistency of the subscales was also high (0.811–0.934; Fabio et al., 2005; Vignoli et al., 2010; Romano et al., 2020). This scale will not be discussed within the scope of the present article.

#### Rehabilitation Goals Achievement

The Goal Attainment Scaling (GAS) was administered to assess the degree of achievement of the rehabilitation objectives identified for each participant. This scaling system represents a mathematical technique used to quantify the identified objectives’ achievement (or nonachievement) and can be used in rehabilitation (Turner-Stokes, 2009). The GAS represents a sensitive measure of patient achievements due to the intervention about a specific therapeutic objective. It has been identified as the most sensitive tool to reflect and measure small changes in patients’ functioning and conditions that otherwise would not be found using standardized measures (Mailloux et al., 2007). Objectives set should follow the SMART principle: They have to be Specific, Measurable, Attainable, Realistic, and Timely (Bovend’Eerdt et al., 2009). This scale and the functional results of this intervention will not be discussed within the scope of the present article.

#### Motor Functioning

The Rett Syndrome Motor Evaluation Scale (RESMES) was used to assess the gross motor functioning of all participants at each evaluation meeting. This tool is a 25-item RTT-specific scale investigating subjects’ gross motor performance across six sections: (a) standing; (b) sitting; (c) transitions; (d) walking; (e) running; and (f) walking up/downstairs. A total score is obtained by summing up the item score with a maximum obtainable score of 82 points (a higher score represents worse gross motor functioning; Rodocanachi Roidi et al., 2019b). RESMES was recently validated on an Italian sample of 60 females with RTT showing optimal inter-rater agreement among clinicians (s×statistic values always>0.70) and strong internal consistency (Rodocanachi Roidi et al., 2019a; Romano et al., 2020). This scale and the motor results of this intervention will not be discussed within the scope of the present article.

#### Parents’ Satisfaction

For quantitative evaluation of parental satisfaction, an *ad hoc* 10-item questionnaire was created to investigate parents’ satisfaction related to program implementation in four areas: (a) adherence and workload (three items); (b) perceived usefulness of the intervention (three items); (c) compliance with the rehabilitation team (two items); and (d) general satisfaction (two items). Questionnaire items were constructed to best represent parents’ satisfaction within the areas of interest. The “Adherence and workload” area referred to the presence or absence in the program implementation; the area of “Perceived usefulness of the intervention” concerned a recognizable improvement of the participant’s motor functional abilities; the “Compliance with the rehabilitation team” area was related to the ability of the researcher to plan and adapt the program to the specific family needs; finally, the “General satisfaction” area was about the parents’ intention to prosecute the program after the project termination. Each item consists of a sentence referred to program implementation or rehabilitative goals achievement. Six sentences were structured in a negative way to support the internal consistency of the questionnaire. The same questionnaire was used in a previous analysis of the satisfaction of parents involved in a similar project (Romano et al., 2021). For each sentence, parents should provide a score of their agreement with the sentence content on a five-point Likert scale from “Completely disagree” (score=1) to “Agree fully” (score=5). Negative sentences were scored in the opposite way: “Completely disagree” (score=5); “Agree fully” (score=1). The average score was obtained for each area from related items scores. A total score ranging from 10 to 50 was calculated by summing up the score given to each item. Within this questionnaire, a higher score corresponded to a better feeling. The questionnaire was received from families by e-mail within 2weeks from the termination of the follow-up evaluation meeting (T4).

To obtain qualitative information about parents’ perspectives concerning their satisfaction related to conducted programs, a semi-structured interview was conducted by EI or MF with participants’ parents at T3 and T4. Open questions were related to program feasibility for both the child and the family, adequacy of program planning, perceived usefulness of the program, and likelihood of continuing the program in the future. Moreover, comments and notes they spontaneously reported within the evaluation meetings and remote supervisions were collected by AR. Parents’ answers to the interviews and comments were transcribed and analyzed, applying thematic analysis to the data from a critical realist perspective, which assumes a socially influenced reality (Willig, 2013). In presenting the analysis, extracts have been edited slightly to ease reading without altering meaning or inference. The thematic analysis process followed the Braun and Clarke (2006) stages, from coding to theme refinement. It was initially inductive and data-driven but later focused on more latent aspects of the data. As the themes presented with the semi-structured interview were not explicitly discussed, the conducted analysis went beyond the semantic content of the data and started to identify the underlying ideas, assumptions, and conceptualizations that are theorized as shaping or informing the semantic content of the data (Braun and Clarke, 2006, 2012). Data were initially subject to multiple readings by AR and ML and coded to identify broad themes in the parents’ perception. From this first thematic mapping, relevant themes were identified by examining codes and coded data by all authors together.

### Statistical Analysis

Pearson rank correlation coefficient was used to explore the relations between parental satisfaction questionnaire total and single area scores and RTT severity level (measured with RARS), level of motor functional abilities (evaluated with RESMES at T1), level of therapeutic goals achievement (measured with GAS at T3 and T4), and parents’ and participants’ ages. The threshold for significance for this analysis has been assumed as *α*=0.05. No correction for multiple comparisons was applied (Armstrong, 2014).

## Results

### Participants and Parents

Among the 42 families involved in the first evaluation (T1), two families (4.8%) did not complete the research protocol. One dropout occurred in the first group and was due to health problems of the participant’s mother that arose during the baseline period. The other dropout happened in the second group. It concerned a family with senior parents living in a rural area with negative external involvement by local healthcare professionals giving the family contradicting advice regarding the suggested habilitation program for the person with RTT and her family. As a result, data analysis involved 40 participants with RTT. Participants’ and parents’ ages, RTT severity level measured with RARS, and motor functional level at recruitment (T1) measured with RESMES were collected in Table 1.

**Table 1:** Descriptive statistics of participants’ and parents’ age, Rett syndrome (RTT) severity level at T1 [measured with Rett Assessment Rating Scale (RARS)] and level of motor functioning at T1 [measured with Rett Syndrome Motor Evaluation Scale (RESMES)].

	Participants’ age (years)	Parents’ age (years)	RARS score at T1	RESMES total score at T1
Mean (SD)	15.7 (9.7)	50.7 (9.6)	/	/
Median	13.3	50.1	67.8	32.5
Max–Min	40.3–2.8	75.1–29.1	82.5–45.5	80–0

/: no data for the cell. SD, standard deviation; RARS, Rett Assessment Rating Scale; RESMES, Rett Syndrome Motor Evaluation Scale; and T1, first evaluation meeting.

At T1, 11 participants were younger than 10 years, 19 were younger than 20 years, and 10 were older than 20 years. Twenty participants (50%) were able to stand independently for more than 1min, and 14(35%) can do it with single or double support given at the hands. Six (15%) participants were unable to sit on a stool without feet and back support for more than 1min. Independent walking for more than 10 steps was possible for 21(52.5%) subjects (one of them could not stand still). Eight participants (20%) showed no scoliosis (seven were young girls under the age of 10years, and one was an adolescent with a high motor functioning level). Twenty-five (62.5%) of them showed some degree of scoliosis (eight showed a mild curve visible only throughout examination in forward bending; for nine participants, the curve was evident in both forward bending and upright position; and, in eight cases, the curve severity prevented the maintenance of the upright position without external support). Seven (17.5%) subjects had undergone spinal fusion surgery. Seven participants (17.5%) attended motor rehabilitative intervention for at least 4h a week. Twenty-eight subjects (70%) attended such interventions between one and 3h per week. Five participants (12.5%) were not involved in any motor rehabilitative treatment. All participants lived at home with their parents. Both the participants and their parents were Caucasian and born in Italy. The participants’ and parents’ daily routines and the number of parents’ working hours varied widely among the participating families. In three families (7.5%), the parents were divorced, and the participants lived with their mothers. In cases where participants lived with both parents, both mother and father participated in the program by doing activities with their daughter. Where the participants lived only with their mother, the program was supported by other family members, such as the participant’s grandparents (two cases) or older siblings (one case).

### Rehabilitation Goals Achievement and Changes in Motor Functioning

As the present article focuses on parental satisfaction and point of view, GAS and RESMES results are only briefly reported.

#### GAS Results

Among 40 subjects, 176 therapeutic goals were identified (mean: 4.4±1.6 goals; range: 1–8 goals). Between identified goals, 28(15.9%) were related to passive limb joints range of motion improvement, 126(71.6%) to functional motor abilities, 15(8.5%) to hand functioning, and seven (4.0%) to general physical health. Among 176 goals, at the end of the intervention phase (T3), 50(24.4%) goals were achieved as expected (GAS score=0), and 95(46.3%) goals were achieved slight (GAS score=+1) or much more than expected (GAS score=+2). Moreover, the level of achievement of 35(19.9%) of these goals continues to improve within the wash-out phase. The average GAS value for all goals for all participants is +0.5±1.0 at post-intervention evaluation (T3).

#### RESMES Results

Within the baseline phase (T1 – T2), nine (22.5%) participants improved their RESMES score, and one (2.5%) worsened it with an average change of −0.2±1.1 points (range: +5 –−4 points). At the end of the intervention (at T3), 34(85.0%) participants (14 from group 1 and 20 from group 2) have reduced RESMES score (suggesting improvements in motor functioning), and no one increased it with an average change of −2.8±2.5 points (range: 0 –−10 points).

### Parents’ Satisfaction

#### Quantitative Data

Quantitative data regarding parents’ satisfaction were assessed through an *ad hoc* questionnaire. On average, of the maximal possible score of 50, a high level of satisfaction was reported by all parents (average total score: 44.1±4.0, range: 50–33). All parents considered the program very helpful for their daughter (average score: 4.8/5±0.4; range: 5–4). Parents who disagreed with the sentence “The program was too hard for my daughter” were 34(85%) while four (10%) agreed with this sentence, and two (5%) reported no opinion related to it (average score: 4.3/5±1.0; range: 5–2). Thirty-eight families (95%) referred that their program was planned correctly (average score: 4.8/5±0.7; range: 5–1) and that it considered the family’s daily routine and lifestyle (average score: 4.8/5±0.6; range: 5–2). Program implementation was reported to be feasible by 39(97.5%) parents (average score: 4.5/5±0.6; range: 5–2). Thirty-two families (80%) approved that the program has changed their daughters’ functional abilities (average score: 4.4/5±1.2; range: 5–1), and 18(45%) referred that the program dramatically changed their daughter functional abilities, while 15 (37.5%) parents disagreed with this sentence (average score: 3.1/5±1.4; range: 5–1). Thirty-eight families (95%) stated that they would continue the proposed activities (average score: 4.6/5±0.7; range: 5–2). These results are described in Table 2.

**Table 2:** Sentences contained in the post-intervention questionnaire with number and percentage of parents’ answers for each score for each sentence.

	I believe that:	Completely disagree	Somewhat disagree	I have no opinion	I agree	Agree fully
1.	The program was very good for my daughter	0–0%	0–0%	0–0%	8–20%	32–80%
2.	The program was too hard for my daughter^*^	20–50%	14–35%	2–5%	4–10%	0–0%
3.	The program was not planned correctly^*^	35–87.5%	3–7.5%	1–2.5%	0–0%	1–2.5%
4.	The program did not consider the child and family’s daily activities^*^	35–87.5%	3–7.5%	1–2.5%	1–2.5%	0–0%
5.	The program was too hard to implement^*^	23–57.5%	16–40%	0–0%	1–2.5%	0–0%
6.	The program did not change my child’s functional abilities^*^	30–75%	2–5%	4–10%	2–5%	2–5%
7.	The program dramatically changed my child’s functional abilities	7–17.5%	8–20%	7–17.5%	11–27.5%	7–17.5%
8.	The program somewhat changed my child’s functional abilities	2–5%	2–5%	6–15%	16–40%	14–35%
9.	I will continue doing the program	0–0%	1–2.5%	1–2.5%	10–25%	28–70%
10.	I will not continue doing the program^*^	37–92.5%	2–5%	1–2.5%	0–0%	0–0%

* Negative sentences;

Scores for positive sentences were assigned as follows: Completely disagree=1; Somewhat disagree=2; I have no opinion=3; I agree=4; and Agree fully=5. Negative sentences were scored the opposite: Completely disagree=5; Somewhat disagree=4; I have no opinion=3; I agree=2; and Agree fully=1.

#### Correlation Analysis

The output of the conducted correlation analysis is summarized in Table 3. A moderate negative correlation emerged between parents’ ages and total satisfaction score and the areas of “Perceived usefulness of the intervention” and “General satisfaction” indicating that older parents were less satisfied with the intervention and less likely to continue the program after the project termination. Consistently, a week negative correlation was found between participants’ age and total satisfaction score and the area of “Perceived usefulness of the intervention” meaning that parents’ of older participants felt the program was less useful. Moreover, the participants’ motor functioning levels showed a weakly negative correlation with the total satisfaction score and a moderate negative correlation with the “Perceived usefulness of the intervention” area denoting that parents of participants with lower motor functional levels were less satisfied with the intervention and perceived it to be less useful.

**Table 3:** Output of Spearman rank correlation coefficient analysis between the satisfaction questionnaire total and individual area scores and RTT severity level (measured with RARS), level of motor functional abilities (evaluated with RESMES at T1), level of therapeutic goals achievement [measured with Goal Attainment Scaling (GAS) at T3 and T4], and parents’ and participants’ ages.

	Satisfaction questionnaire Total score	Adherence and workload area	Perceived usefulness of the intervention area	Compliance with the rehabilitation team area	General satisfaction area
Participants’ age	0.004 (−0.441)^*^	0.192 (−0.211)	0.007 (−0.421)^*^	0.513 (−0.107)	0.005 (−0.439)^*^
Parents’ age	0.018 (−0.371)^*^	0.256 (−0.184)	0.030 (−0.344)^*^	0.326 (−0.159)	0.442 (−0.125)
RARS	0.955 (−0.009)	0.186 (−0.214)	0.746 (0.053)	0.315 (0.163)	0.782 (−0.045)
RESMES at T1	0.022 (−0.360)^*^	0.640 (−0.076)	0.007 (−0.423)^*^	0.769 (−0.048)	0.668 (−0.070)
GAS at T3	0.275 (0.177)	0.191 (0.211)	0.98 (0.004)	0.428 (0.129)	0.815 (0.038)
GAS at T4	0.342 (0.154)	0.965 (0.007)	0.356 (0.150)	0.990 (−0.002)	0.468 (0.118)

* *p*≤0.05;

Each cell contains the relation *p*-value and correlation coefficient (in parenthesis). For “Parents age,” the mean age between the father and the mother ages was calculated. RARS, Rett Assessment Rating Scale; RESMES, Rett Syndrome Motor Evaluation Scale; GAS, Goal Attainment Scaling; T1, First evaluation meeting; T3, Post-intervention evaluation meeting; and T4, Post-wash-out evaluation meeting.

#### Qualitative Data

Within the semi-structured interview, parents spontaneously made important notes and comments related to their participation in this project. Thematic analysis of parents’ answers led to the identification of the following themes: (a) benefits of the program to the child; (b) difficulties of the program to the child; (c) adaptations of the program to the child characteristics; (d) adaptations of the program to child’s and family’s daily routines; (e) changes in child’s functional abilities; (f) continuation of the program after research termination; (g) parents’ perceptional changes; and (h) general reports which emerged at T4 and relate to wash-out phase. The parents’ comments were collected and are presented below divided by themes:

- Benefits of the program to the participant:
  - Family n°2: “The program was very useful for her ability to sit.”
  - Family n°4: “I did not think our daughter could make such important improvements in such a short time, especially related to her kyphosis.”
  - Family n°9: “We have seen an improvement in her posture and muscle strength.”
  - Family n°18: “Since she got used to walking on the treadmill and sitting without a backrest, she is more active throughout the day.”
- Difficulties of the program to the participant:
  - Family n°2: “We were advised to have our daughter lie down supine, which was very difficult for her (and for us to put her in the correct position).”
  - Family n°6: “We were asked to make our daughter stand alone leaning against the railing of the staircase, and we were apprehensive about her scoliosis. It was challenging to do that activity, so we did it less than others.”
  - Family n°12: “Carrying out the program was tough and demanding, but all in all, feasible.”
  - Family n°13: “We always received the support and trust we needed; we were encouraged to do what we could despite the organizational difficulties.”
  - Family n°20: “It was too difficult to get my daughter to use her hands as the stereotypical movements extremely compromise it.”
  - Family n°24: “It was difficult to get her to lie on her back to counteract the kyphosis.”
- Adaptations of the program to the participant’s characteristics:
  - Family n°2: “When they gave the exercises to us, some of them need to be corrected. After talking to the therapist, they were corrected to be well suited to our daughter.”
  - Family n°10: “We felt supported when our daughter showed problematic behavior.”
- Adaptations of the program to the participant’s and family’s daily routines:
  - Family n°17: “The program for my daughter was planned and created especially for her. It was very personalized. We also managed to bring it within the other contexts frequented by the girl (school and rehabilitation center). This project was useful to my daughter and me because I did not think she could do these things.”
  - Family n°18: “If the professionals of the daily center had not been involved, we would not have been able to carry out all the activities.”
- Changes in participant’s functional abilities:
  - Family n°4: “Due to kyphosis, my daughter was more and more bent, but, after all the activities, she has now managed to raise her head, and I find her looking at the sky when we go out.”
  - Family n°5: “We have found a remarkable progress in our daughter who has greatly improved her walking ability being able to take some steps even independently.”
  - Family n°7: “My daughter can now walk independently; before the intervention, we must constantly support her from one arm.”
  - Family n°11: “We believe the program has been very positive for our daughter. We have seen a lot of progress and improvements. With the supervisions via Skype, we have always managed to adapt the program to our needs.”
  - Family n°12: “Now she sits alone on the beach when we go to the sea.”
  - Family n°16 (participants with severe scoliosis): “Even her referring physician has noticed that she remains more straight with her back when she is standing.”
  - Family n°18: “It is now much easier to go for walks with our daughter. She walks much longer and stands on her legs.”
  - Family n°40: “Finally, my daughter can look to her right again.”
- Continuation of the program after research termination:
  - Family n°1: “Seeing the progress that my daughter continues to make motivates us to insist and continue the exercises.”
  - Family n°7: “We have been suggested an excellent way to make our daughter walk more and to make her go up and down stairs; surely we will continue to use them.”
  - Family n°12: “Probably with different rhythms, but we will certainly keep the modalities we have learned.”
- Parents’ perception changes:
  - Family n°12 (relates to a participant who is a constant wheelchair user at program implementation): “We have changed our perspective on the potential of our daughter. Before the program, we had never thought of making her sit without a back for fear of falling. Now we know that before thinking that she cannot do something, it’s worth a try.”
  - Family n°7 (relates to a participant with balance problem at intervention implementation): “Before we did not trust her to walk alone, now we have a strategy to make her more independent.”
  - Family n°13: “The program has been positive for both our daughter and us. It has given us the tools to expand her skills and has opened up prospects for us. Our daughter participated with enthusiasm, and we were given the support and trust necessary to keep us going with the program.”
- General reports emerged at T4 and related to the wash-out phase:
  - Family n°1 (referring to wash-out phase): “We have now incorporated some of the program’s activities into our daughter’s daily life.”
  - Family n°2 (relates to a participant who is a constant wheelchair user at program implementation and referring to wash-out phase): “We think we can no longer stop her sitting alone because she has become good, and she likes it.”
  - Family n°4 (relates to a participant with extreme kyphosis, which continues the intervention after T3): “Last week she managed to lie on her back with her hands behind her head, she had not been able to do it for many years.”
  - Family n°7 (relates to a participant with balance problem at intervention implementation and referring to wash-out phase): “Now she walks alone every day even in our home garden.”
  - Family n°8 (relates to a participant with balance problem at intervention implementation which continues the intervention after T3): “She finally manages to walk downhill alone. A slight slope of course, but she manages to control herself better.”
  - Family n°12 (referring to wash-out phase): “The 3months with all the exercises were tough but very useful. Then we had to bring the activities into our daily lives, and we did not bring everything. Maybe it was more useful for us than for our daughter to develop a new approach. We think it would be useful to repeat this experience in a few months.”
  - Family n°13 (referring to wash-out phase): “It was difficult to maintain the pace of the activities without constant support. For example, in the last few months, we have made her do fewer stairs than within the intervention.”
  - Family n°30 (report collected at T4): “At the end of the intervention, we thought it had been very demanding, now we believe that it would be really useful to be able to repeat this experience after a few months.”

## Discussion

This project aimed to support individuals with RTT and their families by assessing the participant and family’s abilities, constructing an intervention program, and providing remote supervision to program implementation. The focus of the present article is on parents’ satisfaction and perspective. To comply with this goal, the authors first analyzed the satisfaction of parents of girls and women with RTT enrolled in the above-mentioned program from both qualitative and quantitative points of view.

The findings related to rehabilitation goals achievement and motor functioning improvements suggest that this intervention represents an effective way of conducting a remote physical therapy intervention for this population, and this conclusion is supported by previous similar projects (Lotan et al., 2021; Romano et al., 2021). Moreover, it is the authors’ opinion that others with disabilities can be benefited from similar projects. The results suggest that this type of intervention can be applied to individuals with RTT at different severity levels and ages. Moreover, the involved parents spread across a wide range of ages, suggesting that both young and elder parents can participate in this kind of project. The results also support the notion that Skype (in this project) or any other visually enabling application can be used as an effective supervision platform for this type of intervention and can be easily used by parents and caregivers due to its low technological level.

On average, the scores of the parents’ responses to the satisfaction questionnaire revealed a slightly higher level of parental satisfaction than in previous investigation of the authors (Romano et al., 2021). This difference can be explained by the increased experience of the authors in carrying out such intervention programs. Moreover, the results correlate with previous findings (Lotan et al., 2021), where parents were delighted with the program’s adaptability to the person with RTT and her daily routines. Levels of total satisfaction expressed by parents within the present project are coherent with previous reports available in the literature for other populations with disabilities (Assenza et al., 2020; Tanner et al., 2020; Caprì et al., 2021).

Analysis of data collected through the post-intervention questionnaire showed that all parents believed that the intervention was useful for their daughter. The usefulness of the intervention strongly supports parents’ adherence to program implementation (Lillo-Navarro et al., 2019). Four families reported that the program was too difficult for their daughter. The qualitative data suggest that the main difficulties were about parents’ feeling of confidence during exercises performance, a difficulty level which was perceived as too high, and organizational issues. These findings are in line with the existing literature which identified parents perceived self-efficacy, the children’s functional limitations, and parents’ available timetable to adherence and satisfaction with the similar programs (Chappell and Williams, 2002; Rone-Adams et al., 2004; McConnell et al., 2015; Medina-Mirapeix et al., 2017). The present findings also highlight the need for the conducting therapist to be attuned and identify parents’ difficulties in activities performance. In regard to such difficulties, the therapist should dedicate time to properly teach parents the procedures to follow and to help them organize therapeutic activities within their daily routines. Obtained results suggested that, in the present project, except for sporadic cases, the researchers were able to create programs of activities that suited both the therapeutic needs of participants and the daily schedule of their whole families. Adapting the program to the family’s routines and the client’s daily activities is fundamental to support the adherence to the program and to ensure not to increase the families’ stress level (Rone-Adams et al., 2004). Building a therapeutic relationship based on trust and listening between the supervisor and the family is necessary to adapt the program to the emerging needs of clients and their families. Through listening and discussion with caregivers, the elements of difficulty can be identified, and the necessary strategies to cope with them can be implemented. Of importance is the finding that all but one family referred to their intention to continue the program, which means that they found the given suggestions helpful and that the programs were not too hard to implement, coherently with a previous investigation (Romano et al., 2021). The only report of difficulties in the program feasibility came from a single mother with an extremely busy working day. This note again remarks the importance for the therapist to take into consideration the specific timetable of the parents when constructing such a program. Qualitative data related to benefit to the participants suggest that the change in participants’ functional abilities simplified some daily activities of the girls and their families. Moreover, numerous parents stated that the conducted project gave them new strategies to cope with their daughter’s condition and were surprised by the improvements observed.

The large majority of parents declared the intention to continue with the exercises. Two families stated their intention to stop the program. In one case, the parents were of old age and have reported difficulties in carrying out the proposed activity. The other case concerned a girl who began to show pain in her foot during walking activities and in standing position, reducing her motor functional abilities. These data are coherent with the findings that emerged from the correlation analysis which revealed that, in this sample, parental satisfaction (specifically in the area of perceived usefulness of the program) was affected by parents’ and participants’ age and participant’s motor functional level. Despite the broad consensus in the intention to continue the program after the end of the project, the qualitative data suggest that most interviewed parents preferred to continue it at a different pace (less intense). Moreover, at the end of the follow-up phase, the researchers received numerous requests to repeat the project, saying that, however demanding it was, it would be useful to carry it out again and, in general, to alternate periods of development (related to the supervised program) with a period of integration and consolidation of skills and strategies learned (for both participants and their families) within the family’s life, without the constraint of the program. At the same time, several parents reported that they were unable to return to their previous lifestyle as the improvements achieved by their daughters were maintained and consolidated and that they will maintain the good practices learned during the project.

This study has several limitations. The authors did not use a standardized tool to evaluate parental satisfaction. Moreover, the researchers did not collect information related to families’ socioeconomic status, the quality of the relationship between the parents and parents’ occupation, and of daily workload (in hours), which are confounding variables that might affect parents’ satisfaction levels. Finally, the impact of the presence or absence of siblings and other external support availability (e.g., from other family members, nannies, and volunteers) was not assessed. The evaluation of the influence of these variables on the satisfaction level of parents involved in a remote supervised home exercise program should be an integral part of the future investigations.

## Data Availability Statement

The data analyzed in this study are subject to the following licenses/restrictions: The data used for the present study are available in anonymized form from the corresponding author. Requests to access these datasets should be directed to Alberto Romano, alberto.romano01@universitadipavia.it.

## Ethics Statement

The studies involving human participants were reviewed and approved by the Ariel University IRB, Ariel University, Ariel, Israel. Written informed consent to participate in this study was provided by the participants’ legal guardian/next of kin.

## Author Contributions

ML coordinated the project and obtained the funds. ML and AR conducted the participants’ evaluations and wrote the article. AR organized the participants’ evaluations and carried out all remote supervision meetings. EI and MF collected the data which AR analyzed. All authors have read the article and suggested improvements and changes until agreement was reached. All authors are therapists and researchers with experience in treating patients with RTT. Particularly, ML is a world-known researcher, consultant, and physical therapist who have seen more than 600 individuals with RTT across the globe and is working as a clinician with 12 girls with RTT on a weekly basis, and AR is an Italian developmental therapist and researcher who evaluated more than 250 girls and women with RTT around Italy.

## Funding

The International Rett Syndrome Foundation funded the presently described project within the HeART Grant no. 3610.

## Conflict of Interest

The authors declare that the research was conducted in the absence of any commercial or financial relationships that could be construed as a potential conflict of interest.

## Publisher’s Note

All claims expressed in this article are solely those of the authors and do not necessarily represent those of their affiliated organizations, or those of the publisher, the editors and the reviewers. Any product that may be evaluated in this article, or claim that may be made by its manufacturer, is not guaranteed or endorsed by the publisher.
